# Identification of magnetic resonance imaging features for the prediction of unrecognized atrial fibrillation in acute ischemic stroke

**DOI:** 10.3389/fneur.2022.952462

**Published:** 2022-09-13

**Authors:** Chao-Hui Chen, Meng Lee, Hsu-Huei Weng, Jiann-Der Lee, Jen-Tsung Yang, Yuan-Hsiung Tsai, Yen-Chu Huang

**Affiliations:** ^1^Department of Neurology, Chang Gung Memorial Hospital at Chiayi, Chang-Gung University College of Medicine, Chiayi City, Taiwan; ^2^Department of Diagnostic Radiology, Chang Gung Memorial Hospital at Chiayi, Chang-Gung University College of Medicine, Chiayi City, Taiwan; ^3^Department of Neurosurgery, Chang Gung Memorial Hospital at Chiayi, Chang-Gung University College of Medicine, Chiayi City, Taiwan

**Keywords:** ischemic stroke, cryptogenic stroke, cardioembolic stroke, MRI, atrial fibrillation

## Abstract

**Background and purpose:**

The early identification of cardioembolic stroke is critical for the early initiation of anticoagulant treatment. However, it can be challenging to identify the major cardiac source, particularly since the predominant source, paroxysmal atrial fibrillation (AF), may not be present at the time of stroke. In this study, we aimed to evaluate imaging predictors for unrecognized AF in patients with acute ischemic stroke.

**Methods:**

We performed a cross-sectional analysis of data and magnetic resonance imaging (MRI) scans from two prospective cohorts of patients who underwent serial 12-lead electrocardiography and 24-h Holter monitoring to detect unrecognized AF. The imaging patterns in diffusion-weighted imaging and imaging characteristics were assessed and classified. A logistic regression model was used to identify predictive factors for newly detected AF in patients with acute ischemic stroke.

**Results:**

A total of 734 patients were recruited for analysis, with a median age of 72 (interquartile range: 65–79) years and a median National Institutes of Health Stroke Scale score of 4 (interquartile range: 2–6). Of these patients, 64 (8.7%) had newly detected AF during the follow-up period. Stepwise multivariate logistic regression revealed that age ≥75 years [adjusted odds ratio (aOR) 5.66, 95% confidence interval (CI) 2.98–10.75], receiving recombinant tissue plasminogen activator treatment (aOR 4.36, 95% CI 1.65–11.54), congestive heart failure (aOR 6.73, 95% CI 1.85–24.48), early hemorrhage in MRI (aOR 3.62, 95% CI 1.52–8.61), single cortical infarct (aOR 6.49, 95% CI 2.35–17.92), and territorial infarcts (aOR 3.54, 95% CI 1.06–11.75) were associated with newly detected AF. The C-statistic of the prediction model for newly detected AF was 0.764.

**Conclusion:**

Initial MRI at the time of stroke may be useful to predict which patients have cardioembolic stroke caused by unrecognized AF. Further studies are warranted to verify these findings and their application to high-risk patients.

## Introduction

Cardioembolic stroke has been reported to account for about one fifth of all cases of ischemic stroke (1), and it is associated with higher stroke severity and recurrence rate (2, 3). Atrial fibrillation (AF) is the leading cause of cardioembolic stroke (4), and oral anticoagulants are the most effective method to prevent cardioembolic stroke recurrence in patients with AF (5, 6). Therefore, the early diagnosis of AF after stroke is critical to allow for the early initiation of anticoagulant treatment. However, it can be challenging to identify AF, particularly as paroxysmal AF may not be present at the time of stroke. In these situations, 24-h Holter monitoring is the standard method to detect AF, however it is still far from satisfactory. Extended electrocardiogram monitoring may improve the detection rate of AF, however it is expensive and inconvenient, limiting its widespread use in clinical practice. Therefore, a method to accurately identify patients with cardioembolism from unrecognized AF is urgently needed.

Previous studies have used several clinical scales to predict new-onset AF, including CHA2DS2-VASc (congestive heart failure, hypertension, age ≥75 years, diabetes mellitus, prior stroke or transient ischemic attack, vascular disease, age 65–74 years, female) (7). Cohorts for Heart and Aging Research in Genomic Epidemiology-AF (CHARGE-AF) (8), and Electronic Health Record–Based AF (EHR-AF) scores (9). These AF scales have also been shown to be highly associated with cardioembolic stroke (10). However, the use of these scales requires a thorough survey and extensive clinical information to enable calculation of the scores, and their sensitivity and specificity are still far from satisfactory, which limits their usage in the clinic.

Magnetic resonance imaging (MRI) is widely used in clinical practice to identify acute ischemic stroke, and it may be useful to identify cardioembolic stroke from unrecognized AF at the time of stroke. Several infarct patterns on MRI support the diagnosis of cardioembolism, including multiple simultaneous infarcts located in one or more major arterial territories of the anterior and/or posterior circulation (11), single cortical infarction or cortical-subcortical infarct without large artery occlusion (12). In addition, the presence of a susceptibility vessel sign (SVS) in a gradient recalled echo (GRE) MR imaging sequence is associated with erythrocyte-rich thrombus and cardioembolism (13).

In this study, we aimed to evaluate the MRI characteristics associated with newly detected AF among two prospective cohorts of patients with acute ischemic stroke who did not have AF at baseline.

## Materials and methods

### Patients

We analyzed data from two prospective cohorts at Chang Gung Memorial Hospital (14). The first cohort was derived from the Atrial Fibrillation Trial to Evaluate Real-world Procedures for their Utility in helping to Lower Stroke Events (AFTER-PULSE), which compared the detection rate of AF using serial 12-lead electrocardiography vs. 24-h Holter monitoring within 3 months after the index ischemic stroke event, and included elderly patients with no known AF between October 2015 and July 2018 (14). The second cohort was derived from an observational study conducted between January 2014 and September 2017, which evaluated the correlation between left atrial enlargement and new-onset AF among patients with no known AF after their index ischemic stroke event; all patients underwent serial 12-lead electrocardiography and were followed up for 6 months.

**Figure 1:**
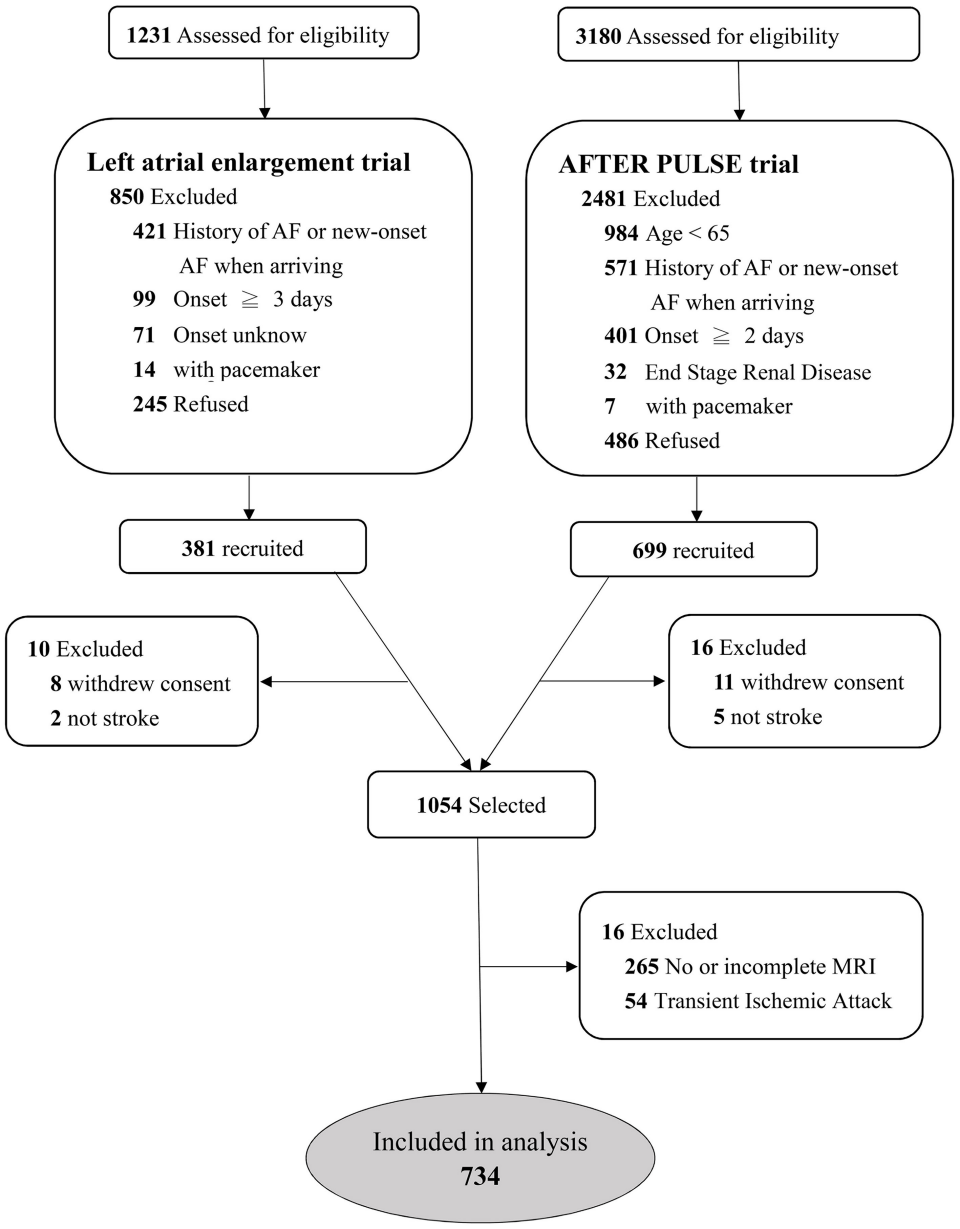
Study flowchart of patient selection.

Among these two cohorts, we selected patients who had undergone MRI within 5 days after the index stroke event and had a visible acute infarction in diffusion-weighted imaging (DWI). A detailed flow chart of patient selection is shown in Figure 1. In both cohorts, data on sex, age, and a medical history of diabetes mellitus, hypertension, hypercholesterolemia, congestive heart failure, prior cerebrovascular disease and prior coronary artery disease were recorded. Systolic and diastolic blood pressure values, blood cell counts and biochemistry data were collected on admission. Neurological deficits were evaluated using the National Institutes of Health Stroke Scale (NIHSS) when the patient arrived at hospital, and the modified Rankin scale at the 90th day.

### MRI protocol and image analysis

All data were collected using a 3 Tesla Siemens Verio MRI system (Siemens Medical System, Erlangen, Germany) or a 1.5-T Philips Gyroscan Intera scanner (Philips Medical Systems, Best, The Netherlands).

Standard sequences included axial DWI, fluid-attenuated inversion recovery images, axial T1- and T2-weighted images, and three-dimensional time of flight angiography covering the extracranial carotid artery and circle of Willis. Patients also received either axial T2^*^-GRE imaging or susceptibility-weighted imaging (SWI). The imaging data were evaluated by two stroke neurologists, who were blinded to the clinical information. If there were any discrepancies in the interpretation of the images, the two readers discussed the data further or consulted a third reader to form a consensus.

### Definition of imaging predictors

#### Classification of DWI patterns

**Figure 2:**
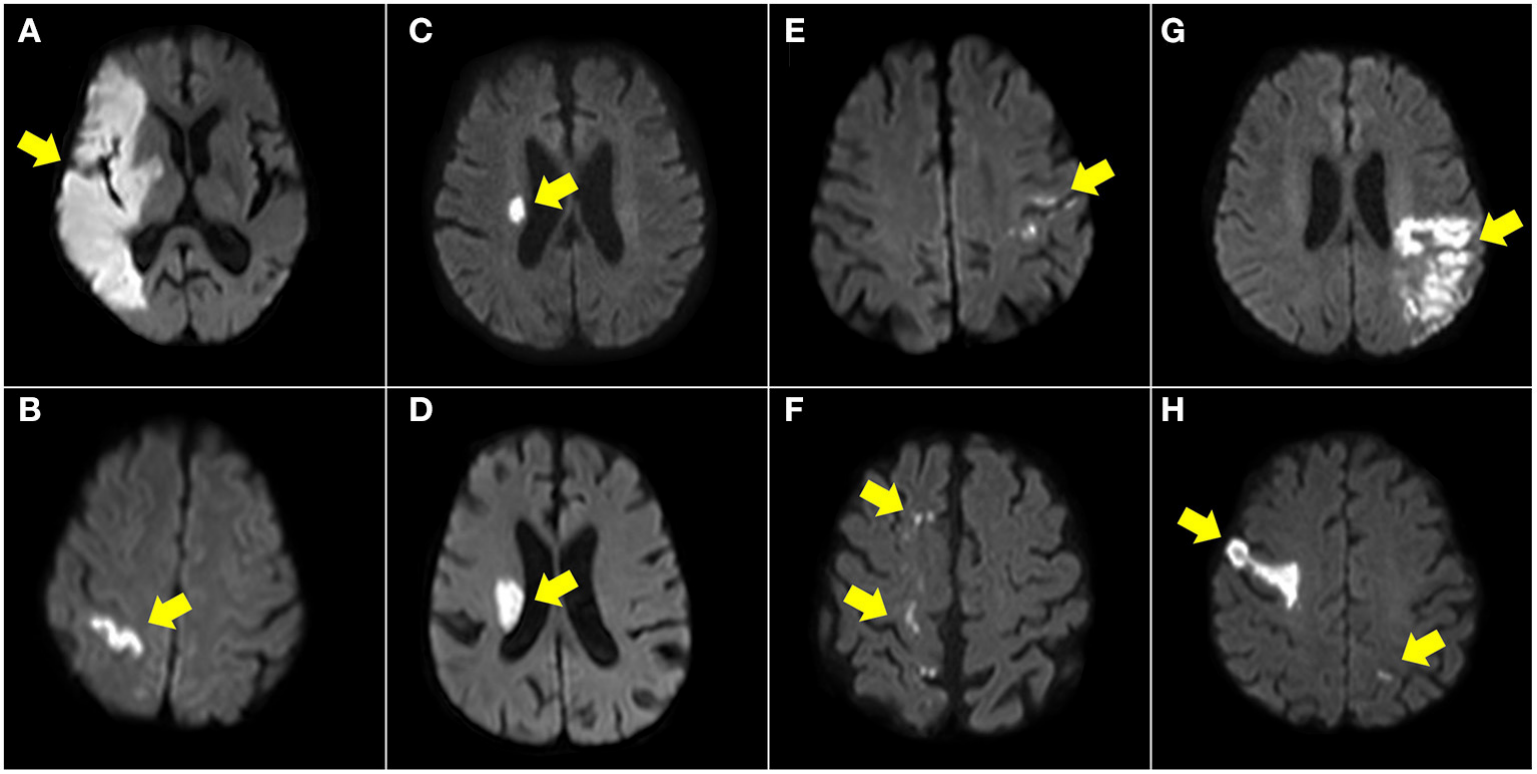
Illustration of DWI patterns in anterior circulation. Territory infarct **(A)**, single cortical infarct **(B)**, single subcortical infarct (<20 mm) **(C)**, single subcortical infarct (>20 mm) **(D)**, small scattered cortical or subcortical infarcts **(E)**, border zone infarcts **(F)**, other cortical and subcortical infarcts **(G)**, and multiple territories **(H)**.

We divided the patients into the following groups based on the observed DWI patterns with reference to previous reports (Supplementary Figure 1) (11, 12, 15, 16), and the illustrated patients are shown in Figure 2 and Supplementary Figure 2.

1. Territorial infarct, involving territories of the internal carotid artery (ICA) or middle cerebral artery (MCA), with at least one division. The DWI pattern should be homogenous, including cortical and subcortical areas. Small separate infarctions in the same vascular territory or in different territories were also classified as territorial infarcts.
2. Single cortical infarct, with a length <30 mm and not involving the subcortical area.
3. Single subcortical infarct (diameter ≤20 mm) in the penetrating artery territories.
4. Single subcortical infarct (diameter ≥20 mm) in the penetrating artery territories.
5. Small scattered cortical or subcortical infarcts, defined as small scattered cortical infarctions with a length <30 mm or multiple subcortical lesions but not restricted to the penetrating artery territories.
6. Border zone infarcts, including internal border zone infarction in the MCA territory or infarction at the MCA-anterior cerebral artery or MCA-posterior cerebral artery cortical border zones.
7. Other cortical and subcortical infarcts, defined as lesions ≥30 mm in one vascular territory but not classified in the patterns above.
8. Multiple territories: multiple infarcts in different vascular territories, including in both left and right ICA territories or in both anterior and posterior circulation territories.

#### Early hemorrhage

**Figure 3:**
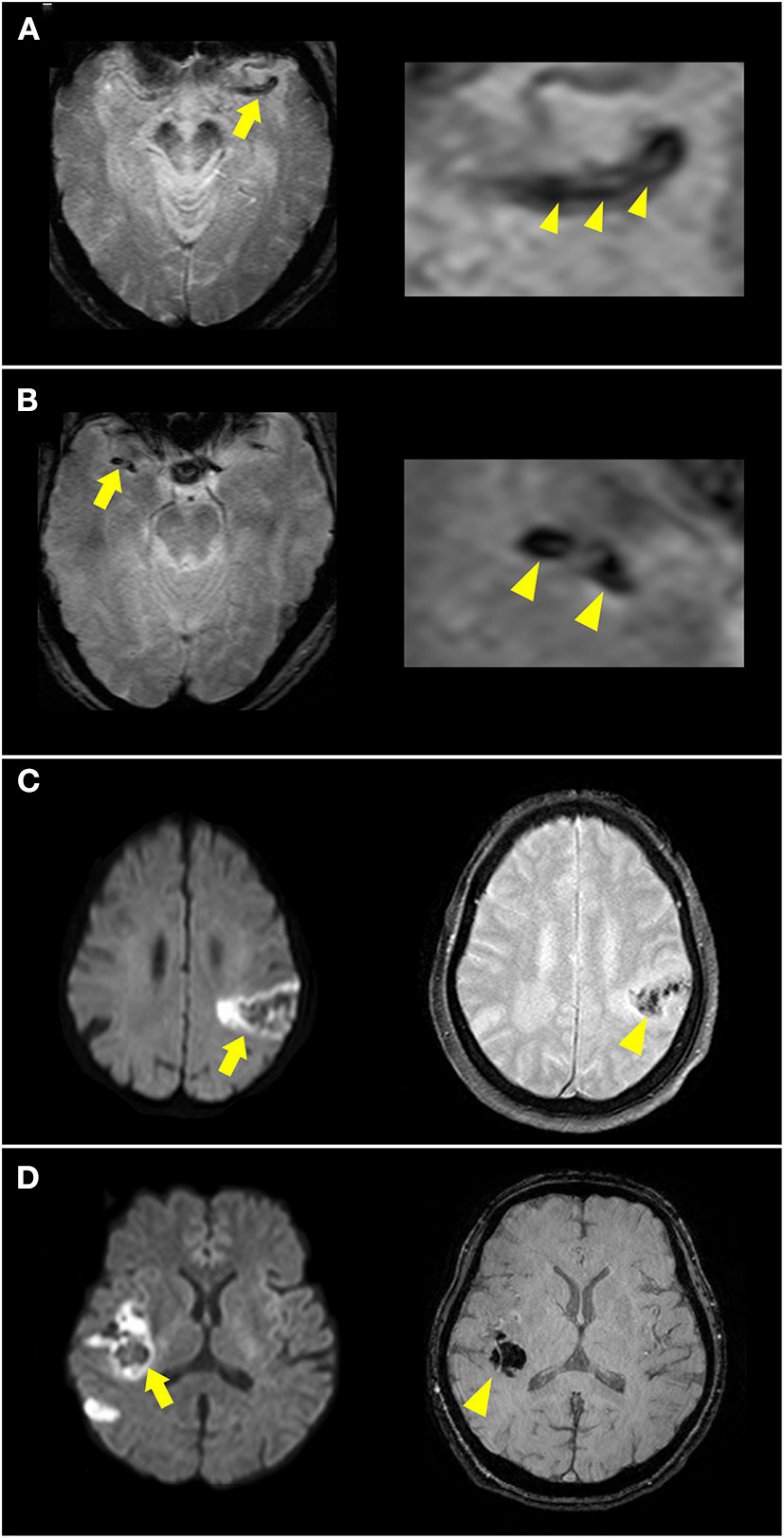
Illustration of susceptibility vessel sign (SVS) and early hemorrhage. **(A)** A hypointense signal was noted at the left occluded middle cerebral artery (MCA) [**(A)**, arrow] in T2*-weighted imaging, with a low-intensity core surrounded by a signal of higher intensity [**(A)** arrowheads], suggesting a 2-layered SVS. **(B)** A homogenous hypointense signal was noted at the right occluded MCA [**(B)**, arrow and arrowheads] in T2*-weighted imaging suggesting SVS. **(C)** DWI showed acute infarction in the left MCA territory with restricted diffusion and hypointense lesions inside (arrow). T2*-weighted imaging showed hypointense signal changes (arrowhead) suggesting hemorrhagic infarction. **(D)** DWI showed acute infarction in the right MCA territory (arrow). Susceptibility-weighted imaging showed a hypointense space-occupying lesion (arrowhead) suggesting parenchymal hemorrhage.

Hemorrhagic transformation after ischemic stroke is related to cardioembolism due to a large core infarction and recanalization. The radiologic appearance of hemorrhagic transformation after ischemic stroke was defined according to the European Cooperative Acute Stroke Study II trial, including hemorrhagic infarction and parenchymal infarction (17). These hemorrhages appeared as hypointense signals within or next to the areas of infarction in SWI or GRE imaging (Figures 3C,D), excluding hemorrhage mimics, such as vessels, mineralization, air-bone interfaces, partial volume artifacts, or microbleeds. Early hemorrhage was defined as any hemorrhagic transformation within 5 days of stroke onset.

#### SVS in SWI and GRE imaging

SVSs from deoxygenated hemoglobin in red clots and imaging markers are highly related to cardioembolism. SVS was defined as a hypointense signal in the symptomatic occlusive vessel on GRE imaging or SWI that was larger than the contralateral arterial diameter (Figures 3A,B).

### Statistical analysis

Descriptive statistics were presented as frequencies, means and standard deviations, or medians and interquartile ranges (IQRs), as appropriate. The Kolmogorov-Smirnov test was used to examine the normality of continuous variables, which were then compared using a Student's *t*-test or Mann-Whitney *U*-test, as appropriate. Categorical data were analyzed using a chi-squared test or Fisher's exact test. All tests were two-tailed, and a *p*-value < 0.05 was considered to indicate a statistically significant difference.

Factors potentially associated with newly-detected AF were evaluated using descriptive statistics. Univariable logistic regression models were used to evaluate candidate variables. Odds ratios (ORs) together with 95% confidence intervals (CIs) were reported, and *p*-values < 0.05 were considered to indicate a statistically significant difference. We built a multivariable regression model based on all potential predictors using forward stepwise selection with *p* < 0.05. Then the “nomolog” package was used to establish a predictive model and generate the nomogram to predict newly detected AF. We also examined discrimination using the C-statistic in our regression model, CHA2DS2-VASc, CHARGE-AF, and EHR-AF scores. Analyses were performed using Stata SE software (version 15.1; StataCorp, College Station, TX).

## Results

A total of 1,054 patients were selected from the two previous trials (Figure 1). After excluding 263 patients without MRI or without complete MRI sequences and 54 patients with transient ischemic stroke, a total of 734 patients were recruited for analysis. The median age of the patients was 72 (IQR 65–79) years, and the median NIHSS score was 4 (IQR 2–6). Among them, 64 (8.7%) patients had AF during the follow-up period; 46 (71.9%) within 14 days, 8 (12.5%) within 15–90 days, and 10 (15.6%) within 90–180 days following stroke onset. Among the 179 patients with embolic stroke of an undetermined source (ESUS), 30 (16.8%) were detected as having AF.

According to the classification of DWI patterns, there were 18 (2.3%) territorial infarcts, 27 (3.7%) single cortical infarcts, 289 (39.4%) single subcortical infarcts with a diameter <20 mm, 61 (8.3%) single subcortical infarcts with a diameter ≥20 mm, 96 (13.1%) small scattered cortical or subcortical infarcts, 58 (7.9%) border zone infarcts, 143 (19.5%) other cortical and subcortical infarcts, and 42 (5.7%) infarcts in multiple territories. Among the included patients, 301 had >50% stenosis or occlusion of the relevant vessels, 40 (5.5%) had hemorrhagic infarcts, and 4 (0.5%) had parenchymal hemorrhage.

Compared to the patients without newly detected AF (Table 1), the patients with AF were significantly older (80 vs. 71 years; *p* < 0.001), included more females (48.4 vs. 34.7%; *p* = 0.028), had a higher rate of ESUS (46.9 vs. 22.2%; *p* < 0.001), lower incidence of diabetes mellitus (31.3 vs. 47.8%; *p* = 0.011), higher incidence of receiving recombinant tissue plasminogen activator (rt-PA) treatment (12.5 vs. 4.0%; *p* = 0.002), and higher incidence of congestive heart failure (7.8 vs. 1.3%; *p* < 0.001). The imaging patterns of territorial infarcts, single cortical infarct, and early hemorrhage were more likely to be associated with newly detected AF, whereas single subcortical infarcts (diameter <20 mm) and border zone infarcts were less likely to be associated with newly detected AF.

**Table 1:** Baseline characteristics, imaging findings and outcomes, and correlations with newly detected atrial fibrillation.

**Characteristics**	**Patients without newly detected**	**Patients with newly detected**	***p***
	**AF**	**AF**	
All patients	670	64	
Age	71 (63–79)	79.5 (73.3–85)	<0.001
Female sex	232 (34.7)	31 (48.4)	0.028
**Stroke information**			
Baseline NIHSS	4 (2–6)	5 (2–8)	0.105
ESUS	149 (22.2)	30 (46.9)	<0.001
Diabetes mellitus	320 (47.8)	20 (31.3)	0.011
Hypertension	519 (77.5)	52(81.3)	0.486
Hypercholesterolemia	259 (38.7)	22 (34.4)	0.501
Coronary artery disease	60 (9.0)	7 (10.9)	0.599
Old stroke	169 (25.2)	11 (17.2)	0.153
Congestive heart failure	9 (1.3)	5 (7.8)	<0.001
Intravenous rt-PA treatment	27 (4.0)	8 (12.5)	0.002
**Imaging patterns**			
Territorial infarcts	12 (1.8)	6 (9.4)	<0.001
Single cortical infarcts	20 (3.0)	7 (10.9)	0.001
Single subcortical infarcts (diameter <20 mm)	272 (40.6)	17 (26.6)	0.028
Single subcortical infarcts (diameter ≥20 mm)	59 (8.8)	2 (3.1)	0.153
Small scattered cortical or subcortical infarcts	86 (12.8)	10 (15.6)	0.527
Border zone infarcts	57 (8.5)	1 (1.6)	0.050
Other cortical and subcortical infarcts	127 (19.0)	16 (25.0)	0.243
Multiple territories	37 (5.5)	5 (7.8)	0.451
Relevant vessel stenosis >50%	279 (41.6)	22 (34.4)	0.247
Susceptibility vessel sign	26 (3.9)	5 (7.8)	0.128
Early hemorrhage	33 (4.9)	11 (17.2)	<0.001

Values presented as n (%) and median (interquartile range).

AF, atrial fibrillation; ESUS, embolic stroke of undetermined source; IQR, interquartile range; NIHSS, national institutes of health stroke scale; rt-PA, recombinant tissue plasminogen activator.

**Figure 4:**
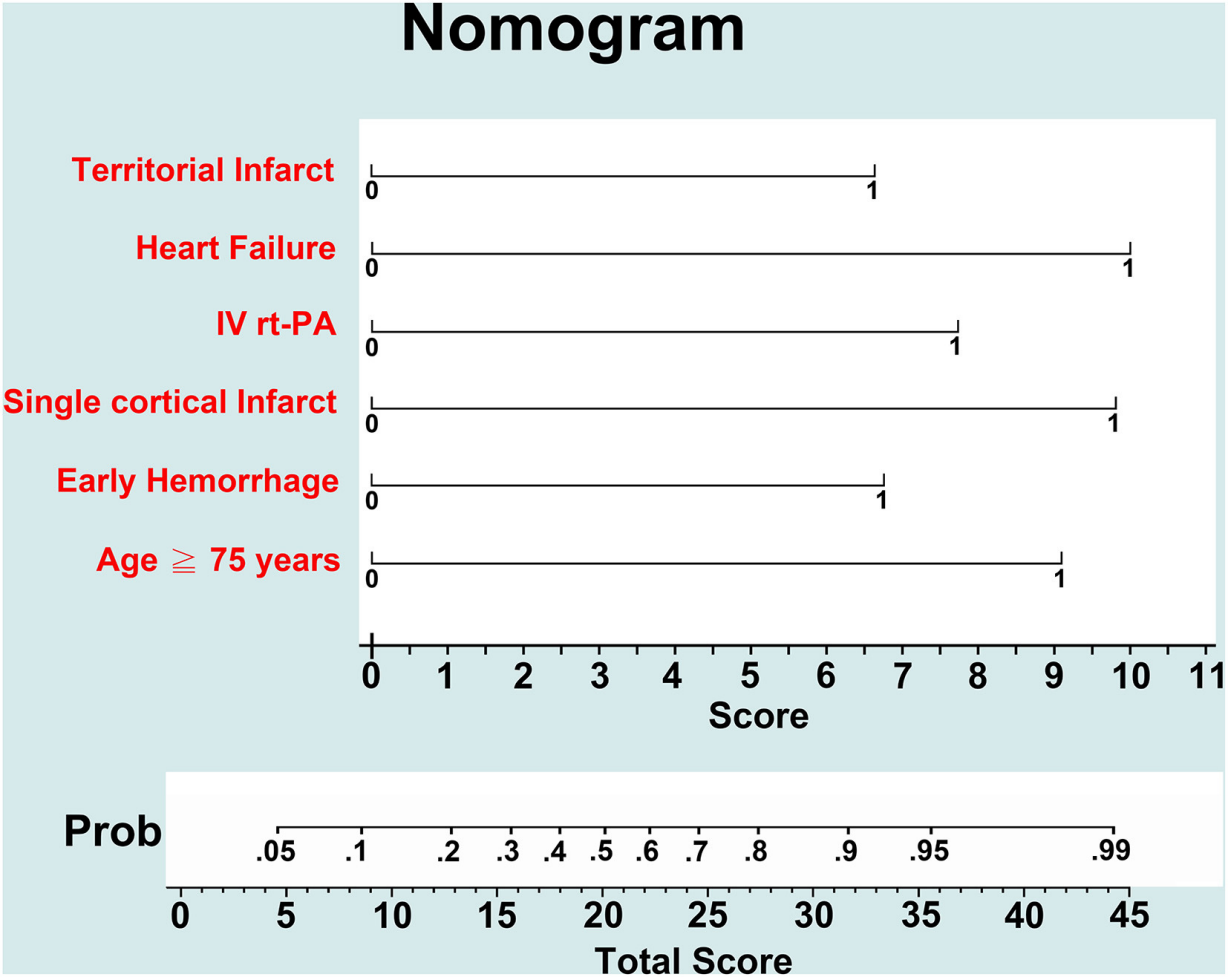
Nomogram of the prediction model for newly detected AF.

In multivariate logistic regression analysis (Table 2), age ≥75 years [adjusted odds ratio (aOR) 5.66, 95% CI 2.98–10.75], receiving rt-PA treatment (aOR 4.36, 95% CI 1.65–11.54), congestive heart failure (aOR 6.73, 95% CI 1.85–24.48), early hemorrhage in MRI (aOR 3.62, 95% CI 1.52–8.61), single cortical infarct (aOR 6.49, 95% CI 2.35–17.92), and territorial infarcts (aOR 3.54, 95% CI 1.06–11.75) were associated with newly detected AF. A nomogram of the prediction model for newly detected AF is shown in Figure 4. The C-statistic of the prediction model for newly detected AF was 0.764 for all patients, and 0.811 for the patients with ESUS. Using CHA2DS2-VASc, EHR-AF and CHARGE-AF score, the C-statistics for the prediction of newly detected AF were 0.551, 0.690, and 0.702, respectively, for all patients.

**Table 2:** Logistic regression for the predictors of newly detected atrial fibrillation in the patients with acute ischemic stroke.

	**Univariate model**			**Multivariate model**		
	**Odds ratio**	**95% CI**	***p***	**Adjusted odds ratio**	**95% CI**	***p***
Age ≧ 75 years	3.93	2.25–6.87	<0.001	5.66	2.98–10.75	<0.001
Female sex	2.05	1.22–3.43	0.006			
NIHSS score	1.06	1.02–1.10	0.006			
Diabetes mellitus	0.50	0.29–0.86	0.013			
Hypertension	1.25	0.65–2.40	0.503			
Hypercholesterolemia	0.86	0.50–1.46	0.572			
Coronary artery disease	1.23	0.54–2.81	0.629			
Old stroke	0.62	0.31–1.21	0.157			
Congestive heart failure	6.22	2.02–19.18	0.001	6.73	1.85–24.48	0.004
Intravenous rt-PA treatment	3.40	1.48–7.84	0.004	4.36	1.65–11.54	0.003
**Imaging patterns**						
Territorial infarcts	5.67	2.05–15.7	0.001	3.54	1.06–11.75	0.039
Single cortical infarcts	3.99	1.62–9.84	0.003	6.49	2.35–17.92	<0.001
Single subcortical infarcts (diameter <20 mm)	0.53	0.30–0.94	0.030			
Single subcortical infarcts (diameter ≧20 mm)	0.33	0.08–1.40	0.134			
Small scattered cortical or subcortical infarcts	1.26	0.62–2.56	0.528			
Border zone infarcts	0.17	0.02–1.25	0.082			
Other cortical and subcortical infarcts	1.43	0.78–2.59	0.245			
Multiple territories	1.45	0.55–3.83	0.453			
Parental vessel stenosis >50%	0.73	0.43–1.25	0.249			
Susceptibility vessel sign	2.13	0.79–5.79	0.137			
Early hemorrhage	4.03	1.92–8.47	<0.001	3.62	1.52–8.61	0.004

CI, confidence interval; NIHSS, national institutes of health stroke scale; rt-PA, recombinant tissue plasminogen activator.

## Discussion

In this study, we demonstrated that specific infarction patterns, including single cortical infarctions, territorial infarctions, and early hemorrhage in MRI independently predicted newly detected AF in patients with acute ischemic stroke. Our findings also challenge the current imaging definition of ESUS, which includes large single subcortical infarctions, as they were not associated with newly detected AF in the current study. Furthermore, the use of additional imaging parameters improved the predictive accuracy for newly detected AF compared to current scales using clinical characteristics (10). A clinical and imaging prediction model may be useful to determine which high-risk patients should receive extended electrocardiogram monitoring. In keeping with previous studies which reported an association between territorial infarcts and cardioembolic stroke, we also found that territorial infarcts were highly associated with newly detected AF in this study (12, 18–20). For acute stroke caused by large vessel occlusion, cardioembolic stroke tends to have less collateral flow compared with atherosclerotic stroke because of the more abrupt perfusion compromise in cardioembolic occlusion (21, 22). As there are fewer leptomeningeal collaterals in cardioembolic stroke, the infarct pattern is unsurprisingly larger, wedge-shaped and homogenous in both cortical and subcortical areas (18, 23). Single or multiple cortical infarcts in either the cerebral or cerebellar cortex have been reported to be related to cardioembolism or AF, suggesting small or fragmented cardio-emboli (24–26). However, only single cortical infarcts were associated with newly detected AF in our study, and multiple cortical infarcts were not. The reason for this finding could be that multiple cortical infarcts are also related to artery-to-artery embolism from atherosclerotic plaques of steno-occlusive vessels, whereas single cortical infarcts are more likely to be related to the cardioembolism (26).

Multiple simultaneous infarcts in multiple territories have been reported to be more prevalent in patients with an acute ischemic stroke of cardioembolic origin (27) and in patients with occult AF in ESUS (28). However, in the current study, there was no significant difference in infarctions in multiple territories between the patients with or without newly detected AF. The reason for this may be that we excluded patients with a documented risk of cardioembolism and included patients with small vessel occlusion. Therefore, only 5.2% of the patients presented with multiple circulation infarcts in our study. The lack of significant correlation between multiple simultaneous infarcts in multiple territories and AF may also be due to the small sample size. In addition, we did not exclude patients with large atherosclerotic occlusion, which may have also led to multiple circulation infarcts, such as in bilateral anterior cerebral arteries from unilateral ICA or a fetal type posterior cerebral artery. Despite these limitations, our findings are close to clinical practice and provide valuable information that the presence of multiple simultaneous infarctions is not as useful as previously assumed for the prediction of unrecognized AF.

The infarction pattern of single subcortical infarcts with a diameter <20 mm, commonly called lacunar infarcts, was less likely to be associated with newly detected AF in this study, which is comparable with previous reports (12, 27). A single subcortical infarct with a diameter ≥20 mm still showed a non-significant trend toward identifying the absence of AF. The major reason for this may be that larger single subcortical infarcts are usually attributed to branch atheromatous disease caused by atherosclerotic plaques involving parent artery perforators and less to cardioembolic occlusion (29). Nevertheless, large single subcortical infarcts with a diameter ≥20 mm are classified as ESUS according to imaging criteria. Since their pathologies favor an atherosclerotic origin rather than cardioembolism, our findings provide evidence against the current definition of ESUS, which includes single subcortical infarcts ≥20 mm (30). In the RE-SPECT ESUS and NAVIGATE ESUS trials, rivaroxaban and dabigatran were not shown to be beneficial in preventing recurrent ESUS. A possible reason for this finding may be the heterogeneity of the recruited patients (31). In our study, 34% (64/179) of the patients with ESUS had a single subcortical infarction with a diameter ≥20 mm. Therefore, it may be reasonable to remove single subcortical infarcts with a diameter ≥20 mm from the imaging definition of ESUS, and instead use a new definition involving MRI imaging.

Both internal border zone infarction and cortical border zone infarction are usually associated with hemodynamic failure and microembolization from steno-occlusive disease or sometimes systemic hypotension (32, 33). When border zone infarction is combined with stenosis or occlusion of relevant vessels, it is usually presumed and classified to be a large atherosclerotic artery stroke in clinical practice. Hence, it is no surprise that the pattern of border zone infarction was less likely to be associated with newly detected AF in our study. However, time of flight angiography in MRI could not clearly distinguish between atherosclerotic and cardioembolic occlusion, so that occlusion or stenosis >50% of relevant vessels failed to predict newly detected AF. Although SVS with specific morphologies has been shown to be associated with cardioembolic stroke (34, 35), the trend was not statistically significant in our study, probably due to the low number of patients (4.2%) with SVS. Further studies with a larger sample size and a precise definition of SVS are warranted to test its efficacy for the prediction of newly detected AF.

Cardioembolic stroke, especially that caused by AF, is strongly associated with hemorrhagic transformation after acute ischemic stroke, probably due to the inherent characteristics of large infarction core and early recanalization (36–38). T2^*^-weighted GRE and SWI are sensitive methods of detecting early hemorrhagic transformation after acute ischemic stroke (39, 40). In our study, early hemorrhagic transformation in MRI was an independent predictor for newly detected AF.

In addition to specific imaging patterns, we also found that certain clinical characteristics, including female sex, age ≥75 years, and heart failure, were associated with an increased risk of newly detected AF. These factors are also included in the CHA_2_DS_2_-VASc score, which is used to predict the risk of stroke in individuals with AF, and predict the risk of AF in individuals without AF (41, 42). The CHARGE-AF and EHR-AF scores have been used to predict new-onset AF in population-based cohorts and to predict cardioembolic stroke at the time of stroke (9, 10, 43). AF has also been reported to account for ~25% of patients receiving thrombolytic therapy because of worse stroke severity (44, 45). Therefore, the patients receiving intravenous rt-PA treatment were related to newly detected AF. The prediction model in our study was robustly associated with newly detected AF and could moderately discriminate occult cardioembolism from unknown paroxysmal AF. A prediction model including MRI information may improve the accuracy of predicting newly detected AF at the time of stroke.

Compared with the NAVIGATE and RESPECT ESUS trials, the definition of cardiac embolism in the ATTICUS trial included additional risk factors (46). Despite the higher rate of newly detected AF (23%), the trial still failed to demonstrate the efficacy of apixaban in ESUS patients. Moreover, apixaban treatment was not superior to “aspirin with the switch to apixaban in case of AF detection by mandatory cardiac monitoring” in preventing new ischemic lesions during follow-up. This may emphasize the importance of extended electrocardiogram monitoring in patients with ESUS, and our prediction model may be useful to identify high-risk patients.

There are several limitations to this study. First, we presumed that the specific infarction patterns were related to cardioembolism due to unknown paroxysmal AF, however other etiologies of embolic stroke were not explored, such as patent foramen ovale, valvular heart disease, aortic arch atheroma, cancer-associated coagulopathy, etc. Second, some patients only received serial 12-lead electrocardiography and were followed up for 3 months. The incidence of newly detected AF may therefore be underestimated in our study, even though the AF detection rate of 8.7% in our ESUS patients is comparable to the 8.9% reported in a previous trial using an insertable cardiac monitor for 6 months (47). The small sample size and the fact that we did not use prolonged cardiac monitoring may mean that we missed potential predictors for unknown paroxysmal AF. Third, we did not recruit patients with endovascular thrombectomy, so the imaging patterns cannot be applied in these patients. Fourth, our study has the inherent drawbacks of selection and detection bias, since one selected cohort excluded patients <65 years old and end-stage renal disease, and we only selected patients who underwent MRI and had a milder stroke severity. Despite these limitations, our study still provides valuable information which may improve the prediction of newly detected AF after acute ischemic stroke.

## Conclusion

In addition to clinically known risk factors for AF, our study revealed that MRI at stroke onset provides critical clues for the prediction of newly detected AF, including single cortical infarcts, territorial infarcts and early hemorrhage. Future studies are warranted to verify this new prediction model and to assess whether the identification of AF can be enhanced to improve outcomes after acute ischemic stroke.

## Data availability statement

The original contributions presented in the study are included in the article/Supplementary material, further inquiries can be directed to the corresponding author.

## Ethics statement

The protocols of these studies were approved by the Institutional Review Board of Chang Gung Memorial Hospital (103-7597B and 104-9611C). The patients/participants provided their written informed consent to participate in this study.

## Author contributions

Y-CH interpreted data, analyzed imaging, and revised the manuscript. C-HC analyzed imaging and drafted the manuscript. ML designed the study and interpreted data. J-DL and J-TY acquired data. Y-HT analyzed the imaging. H-HW conducted the statistical analyses. All authors reviewed the manuscript and gave final approval to the version to be published.

## Funding

This study was supported by Chang Gung Memorial Hospital research grants (CMRPG6J0361, CMRPG6J0362, and CORPG6G0223).

## Conflict of interest

The authors declare that the research was conducted in the absence of any commercial or financial relationships that could be construed as a potential conflict of interest.

## Publisher's note

All claims expressed in this article are solely those of the authors and do not necessarily represent those of their affiliated organizations, or those of the publisher, the editors and the reviewers. Any product that may be evaluated in this article, or claim that may be made by its manufacturer, is not guaranteed or endorsed by the publisher.
