# Supplementary material for: Identification of magnetic resonance imaging features for the prediction of unrecognized atrial fibrillation in acute ischemic stroke
Source: Front Neurol. 2022 Sep 13;13:952462. doi: 10.3389/fneur.2022.952462 (PMC9513827; doi:10.3389/fneur.2022.952462)
Supplement: Supplementary file 1 [file Data_Sheet_1.pdf]

Supplemental Material

Identification of magnetic resonance imaging features for the prediction of unknown atrial fibrillation in acute ischemic stroke

Chao-Hui Chen[1], Meng Lee[1], Hsu-Huei Weng[2], Jiann-Der Lee[1], Jen-Tsung Yang[3], Yuan-Hsiung Tsai[2] and Yen-Chu Huang[1]*

Department of Neurology[1], Department of Diagnostic Radiology[2], and Department of Neurosurgery[3], Chang Gung Memorial Hospital at Chiayi, Chang-Gung University College of Medicine, Taiwan

Table S1.

Baseline characteristics, imaging findings and outcomes in two cohorts

Figure S1.

Study flowchart for the classification of DWI patterns.

Figure S2.

Illustration of DWI patterns in posterior circulation. Single cortical infarct (A), single subcortical infarct (<20mm) (B), single subcortical infarct (>20mm) (C), small scattered cortical or subcortical lesions (D), border zone infarcts (E), other cortical and subcortical infarcts (F) and multiple territories (G).

Table S1.

Baseline characteristics, imaging findings and outcomes in two cohorts

**Characteristics**	**AFTER PULSE cohort**	**Left Atrial Enlargement cohort**	***p***
Total patients	368	366	
Age	75 (69–81)	65 (56–76)	<0.001
Female gender	139 (37.8)	123 (33.6)	0.239
**Stroke information**			
Baseline NIHSS	4 (2–7)	4 (2–6)	0.028
ESUS	81 (22.0)	98 (26.8)	0.133
Diabetes mellitus	168 (45.7%)	172 (47.0)	0.715
Hypertension	293 (79.6)	279 (76.2)	0.268
Hypercholesterolemia	134 (36.4)	154 (42.1)	0.116
Coronary artery disease	32 (8.7)	36 (9.8)	0.594
Old stroke	76 (20.7)	103 (28.1)	0.018
Congestive heart failure	7 (1.9)	7 (1.9)	0.992
Intravenous rt-PA treatment	17 (4.6)	18 (4.9)	0.850
Newly-detected atrial fibrillation	40 (10.9)	24 (6.6)	0.038
**Imaging patterns**			
Territorial infarcts	11 (3.0)	7 (1.9)	0.346
Single cortical infarct	9 (2.4)	18 (4.9)	0.075
Single subcortical infarct (diameter<20mm)	146 (39.7)	143 (39.1)	0.867
Single subcortical infarct (diameter≧20mm)	34 (9.2)	27 (7.4)	0.361
Small scattered cortical or subcortical infarcts	49 (13.3)	47 (12.8)	0.849
Border zone infarcts	30 (8.2)	28 (7.7)	0.801
Other cortical and subcortical infarcts	71 (19.3)	72 (19.7)	0.897
Multiple territories	18 (4.9)	24 (6.6)	0.331
Relevant vessel stenosis >50%	159 (43.4)	142 (38.9)	0.213
Susceptibility vessel sign	16 (5.1)	15 (4.2)	0.600
Early hemorrhage	20 (6.2)	24 (6.6)	0.823

Values presented as n (%) and median (interquartile range).

Abbreviations: AF, atrial fibrillation; ESUS, embolic stroke of undetermined source; IQR, interquartile range; NIHSS, National Institutes of Health Stroke Scale; rt-PA, recombinant tissue plasminogen activator.

Figure S1. *Study flowchart for the classification of DWI patterns.*

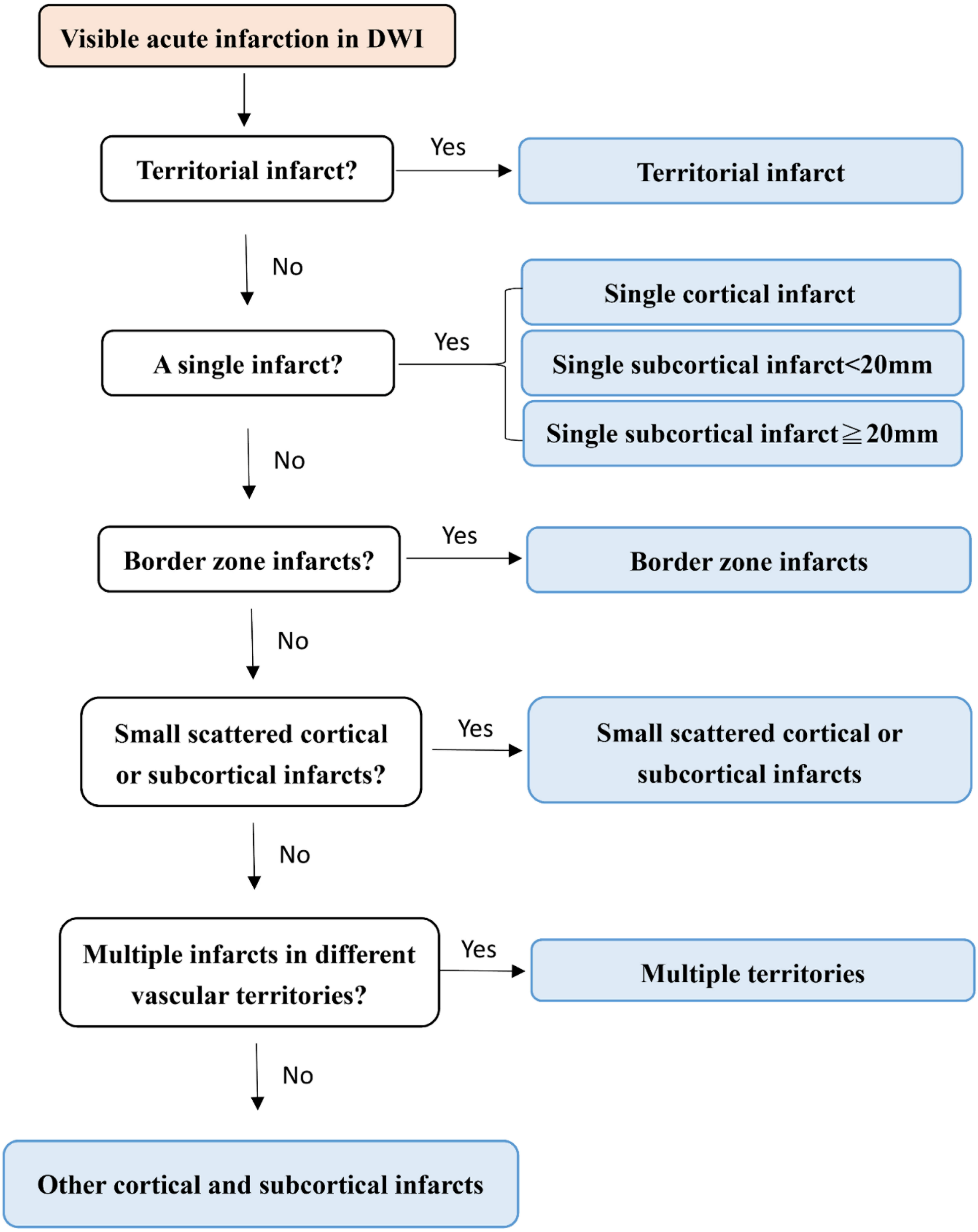

Figure S2.

Illustration of DWI patterns in posterior circulation.

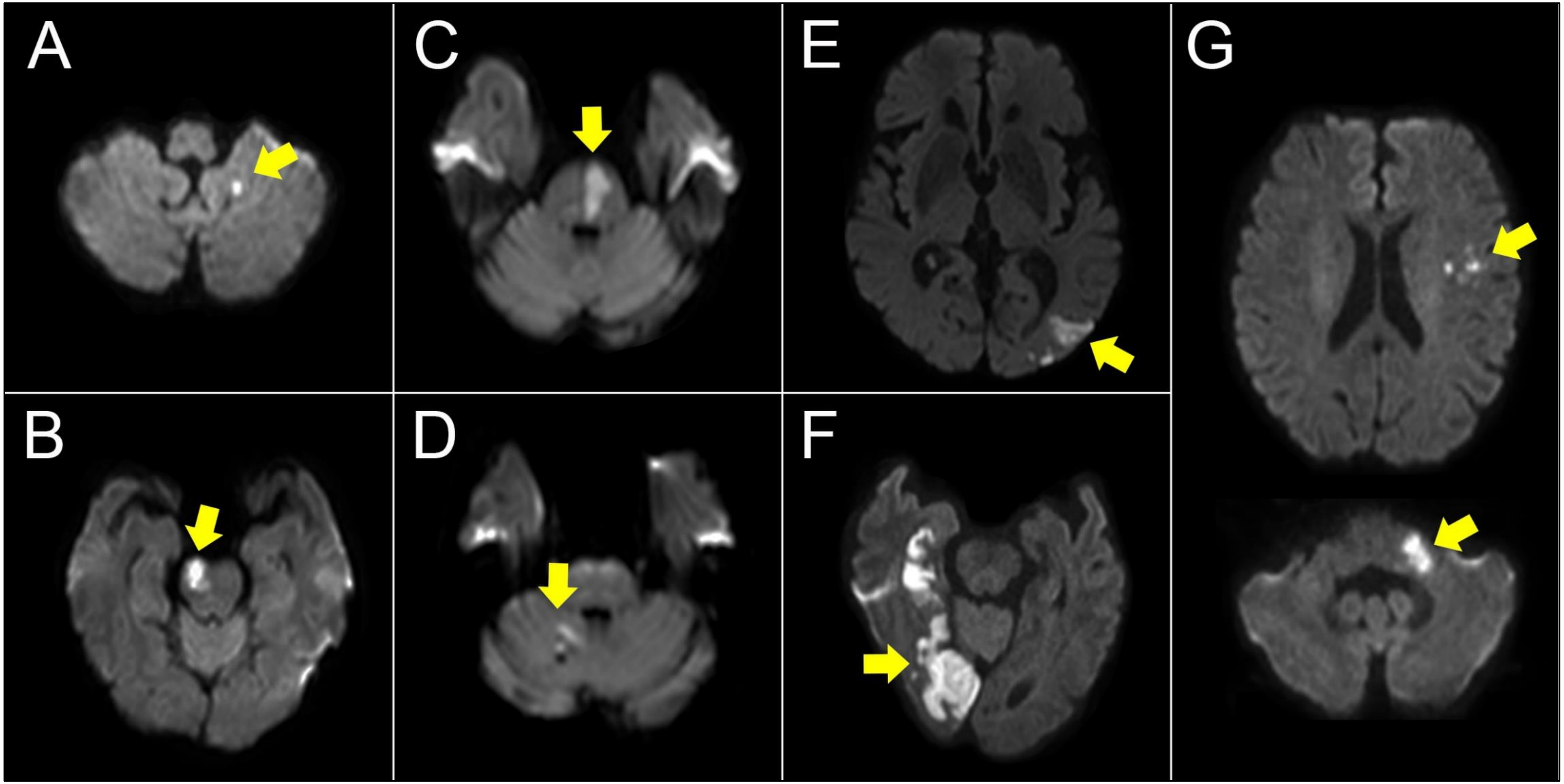

Single cortical infarct (A), single subcortical infarct (<20mm) (B), single subcortical infarct (>20mm) (C), small scattered cortical or subcortical lesions (D), border zone infarcts (E), other cortical and subcortical infarcts (F) and multiple territories (G).
